# A deep image classification model based on prior feature knowledge embedding and application in medical diagnosis

**DOI:** 10.1038/s41598-024-63818-x

**Published:** 2024-06-09

**Authors:** Chen Xu, Jiangxing Wu, Fan Zhang, Jonathan Freer, Zhongqun Zhang, Yihua Cheng

**Affiliations:** 1 School of Computer Science, Fudan University, Shanghai, China; 2 School of Computer Science, University of Birmingham, Birmingham, UK

**Keywords:** Image classification, Deep learning, Self-attention mechanism, Prior feature knowledge embedding, Small sample set modeling, Computer science, Image processing

## Abstract

Aiming at the problem of image classification with insignificant morphological structural features, strong target correlation, and low signal-to-noise ratio, combined with prior feature knowledge embedding, a deep learning method based on ResNet and Radial Basis Probabilistic Neural Network (RBPNN) is proposed model. Taking ResNet50 as a visual modeling network, it uses feature pyramid and self-attention mechanism to extract appearance and semantic features of images at multiple scales, and associate and enhance local and global features. Taking into account the diversity of category features, channel cosine similarity attention and dynamic C-means clustering algorithms are used to select representative sample features in different category of sample subsets to implicitly express prior category feature knowledge, and use them as the kernel centers of radial basis probability neurons (RBPN) to realize the embedding of diverse prior feature knowledge. In the RBPNN pattern aggregation layer, the outputs of RBPN are selectively summed according to the category of the kernel center, that is, the subcategory features are combined into category features, and finally the image classification is implemented based on Softmax. The functional module of the proposed method is designed specifically for image characteristics, which can highlight the significance of local and structural features of the image, form a non-convex decision-making area, and reduce the requirements for the completeness of the sample set. Applying the proposed method to medical image classification, experiments were conducted based on the brain tumor MRI image classification public dataset and the actual cardiac ultrasound image dataset, and the accuracy rate reached 85.82% and 83.92% respectively. Compared with the three mainstream image classification models, the performance indicators of this method have been significantly improved.

## Introduction

Image classification is an important research topic in computer vision. It predicts the category of input images from a set of preset categories based on the unique local and global features extracted from the image^1^. Presently, deep learning stands as the predominant approach for image classification, with Convolutional Neural Networks (CNNs) being extensively employed for image feature extraction. The local connectivity and translation invariance inherent in CNNs make them well-suited for tasks involving image feature representation. Furthermore, it effectively enhances the capacity to extract and represent distinctive image features by integrating multi-scale and attention mechanisms into learning process^2–4^. Despite these advancements, practical applications of image classification have several challenges, including the presence of insignificant morphological and structural features, substantial variations in target morphology and size within images, strong correlations and low signal-to-noise ratio. Addressing these issues requires strategies to emphasize the importance of unique image features, prevent the loss of information related to small target features, and optimize intra-class similarity while minimizing inter-class similarity of image features^5–7^. For example, when deep learning methods are applied to medical image classification with amorphous structural boundaries, their effectiveness exhibits significant variability^8^, and there are issues such as insufficient attention to local key features and potential saliency features being ignored^9^. Additionally, deep learning relies on extensive, comprehensive datasets. However, it is often difficult to obtain large-scale and complete datasets in many applications due to factors such as non-repeatability of certain event processes, fewer occurrence of some scenarios and high sampling costs. Recently, few-shot learning is used to handle the insufficient data problem. It uses methods such as model initialization^10^, transfer learning^11^, and matching networks^12^. However, these methods necessitate meet certain conditions, including the requirement for the initial dataset and the target small-sample set to share the same or similar distribution, clear correlation information between two, or the universal applicability of feature extractors^13,14^. These conditions still present difficulties in unknown or cognitive limited research fields. While data augmentation is commonly used, its ability to enhance the diversity of image features is limited, thus constraining its effectiveness in reducing the upper bound of generalization error^15^. Consequently, this approach carries structural risks and may exhibit insufficient generalization ability when modeling a small dataset. As a result, for complex image classification tasks, there are still challenges on extracting features, improving the identifiability of features, and modeling small sample sets^16,17^.

This paper introduces a method for embedding diversity prior category feature knowledge to alleviate above issues. The main contributions are as follows:

- We propose a deep learning-based image classification method using ResNet50 for image feature extraction and RBPNN for embedding diversity prior class feature knowledge. It designed targeted functional modules for image characteristics in mechanism, which can extract local and global features unique to images at multiple scales, strengthen the role of category prior feature knowledge in classification, improve the limitations of existing neural networks that can only form convex decision regions, thereby enhances image classification accuracy.
- The proposed classification method is based on RBPNN for prior class feature embedding, which can achieve structural and data constraints on the model, reduce structural risks in small sample set modeling and the requirements for sample set completeness. In terms of mechanism, it is suitable for modeling small sample sets of medical images.
- The feature extraction method is based on multi-scale and mixed self-attention mechanisms, which can finely depict local and global structural features of images, effectively improving the recognition ability of features in images with insignificant morphological structural features and low signal-to-noise ratio. Meanwhile, selection method of typical prior sample features for categories can maintain feature diversity and improve the model robustness.
- Channel cosine similarity attention is used to measure the similarity between input image features and diversity category feature templates. Based on the nature of radial basis kernel transformation, it can transform complex image classification problems into solving the nearest neighbor problem in the feature space, that is, by comparing the distance between the target image features and the center of each pattern subclass for classification, in order to maintain the algorithm’s generalization ability.

## Related works

### Image multi-scale feature extraction and attention mechanism

For multi-scale feature extraction of images, due to the significant influence of receptive fields on the extraction and representation of local to global features, researchers have designed various multi-scale model architectures to extract image features through layer by layer such as AlexNet^18^, VGGNet^19^, GoogLeNet^20^, and ResNet^21^. The Inception Network^22^ uses a multi-scale fusion structure with parallel branches, which can obtain features of different receptive fields at the same level, then transfer them to the next layer after fusion. The serial multi-scale feature structure represented by a fully convolutional network (FCN)^23^ achieves the fusion of features at different depths and levels through skip-connections, improving the resolution ability of high similarity images. The SSD^24^ uses a multi-scale feature fusion structure to extract features from feature maps at different levels using different stripes to detect features of targets of different sizes. SSH^25^ divides high-resolution feature maps into multiple branches, detects targets of different scale sizes separately through each branch. PSPNet^26^ uses a receptive-field control method to compress the model through pooling operations of different sizes. Big-little Net^27^ processes image resolution information at different scales. It uses fewer or larger convolutional channels for branches with high or low resolution, in order to fully utilize channel information. Liu et al.^28^ proposed an adaptive scale deep model that establishes the optimal size set and adaptive learning mechanism to obtain the optimal solution. Chen et al.^29^ proposed a dual branch Transformer to combine image patches with different sizes and extract multi-scale feature representations to produce more prominent image features.

For the study of attention mechanisms, various attention mechanisms and their improved forms have been proposed. These methods consider varying contributions of feature information from different regions to image classification tasks, emphasizing regions with higher task relevance for cognitive attention^30^. Spatial attention^31^ uses a masking mechanism, which obtains weight masks through network training to identify key feature regions in the image. The representative model is the Spatial Transformer Network (STN) proposed by Google^32^. It leverages positioning networks and grid generators to output spatial attention weight masks, adjust the attention to feature map regions with different depth. The channel attention mechanism^33^ models the importance of each functional channel of input features, enhancing or weakening the feature information of different channels according to different tasks. The representative model is the squeeze-and-excitation network (SENet)^34^, which automatically captures the significance of each feature channel through the SE module, enhance or suppress channel features which are useful or irrelevant for the current task. Self- attention^35^ has been widely studied in recent years. Cheng et al.^36^ proposed a modular combination attention block ResGANet, which can capture feature dependencies in images in two independent dimensions of channel and space. By stacking attention blocks through ResNet, ResGANet model can be directly used for image classification. He et al.^37^ proposed a self-supervised learning method MoCo (Momentum Contrast) based on attention mechanism for training image representation learning models. By building a contrastive learning framework to utilize large-scale unlabeled image data for unsupervised learning, the model learns discriminative feature representations by maximizing the similarity between query images and positive samples and minimizing the similarity with other images. Oquat et al.^38^ designed a novel visual model training method DINO v2, which trains the network by setting two objective functions: image level objective and patch level objective. It adopts attention mechanism and self-supervised learning to achieve better classification results.

### Approach in small sample set modeling

Researchers have studied the problem of modeling small sample sets in image classification and proposed many effective strategies and methods. Based on model initialization, Ravi et al.^39^ proposed an optimization algorithm using an LSTM-based meta learner to train another learner with a small number of training samples to adapt to the needs of scene updates. Nakamura et al.^40^ proposed an initialization model that sets a lower learning rate when retraining on small sample categories, and improves the network’s generalization ability by making appropriate adjustments to network. For study of data augmentation, Wang et al.^41^ used additional unsupervised meta training to allow multiple top-level units to learn a large amount of unlabeled data from real images to obtain more general features, facilitating the extension of the features learned by the network to other categories. Boney et al.^42^ proposed a semi-supervised learning method using Model Independent Meta Learning (MAML) strategy. The model uses unlabeled data to adjust the embedding function parameters and labeled data to adjust parameters of the classifier. Hong et al.^43^ proposed a labeled data augmentation method for Fusing and filling Generative Adversarial Network (F2GAN), which utilizes a small group of images to generate more realistic and diverse images. Based on transfer learning, Sun et al.^44^ proposed a Meta Transfer Learning (MTL) method to learn the weights of transfer deep neural networks, which improves learning efficiency by introducing a hard task meta batch processing scheme. Yu et al.^45^ proposed a transfer learning framework based on semi-supervised small sample learning. Based on pre-trained feature extractor of the base class data, the extractor is used to initialize classifier weights from new class. Finally, a semi-supervised learning method is used to update the model in deeper level to achieve data transfer learning. Matching network^46^ is a neural network model that introduces attention mechanism, which can achieve end-to-end training by combining feature extraction, differentiable models, and cosine similarity. Cai et al.^47^ proposed a Memory Matching Network (MM-Net) to explore the training process. Firstly, the image features extracted from the support set and the corresponding category labels are stored as key value pairs in the memory module. Then, the query image is compared with the feature information in the memory module, and the category with the highest similarity is selected as the query image category. Singh et al.^48^ proposed a gradient based meta learning model MetaMed, which used image enhancement techniques such as MixUp, CutOut, and CutMix to regularize the model during the training phase, effectively overcoming overfitting problems and achieving good results.

### Image classification model

Many state-of-the-art classification models have been proposed based on practical application requirements. Zhang et al.^49^ proposed a Synergic Deep Learning (SDL) model consisting of multiple pre-trained deep convolutional neural networks (DCNN) and synergistic networks. Each DCNN learns image representation and classification, and connects the learned image representations as inputs to the synergistic network to predict the category of the input image. Dosovitskiy et al.^50^ improved the Transformer for natural language processing and proposed a Vision Transformer (ViT) model for image classification. In this model, the whole image is divided into several small image blocks, the linear embedding sequence of these image blocks is taken as the input of Transformer network, and image classification is performed based on Supervised learning. Abdar et al.^51^ proposed two feature fusion methods: direct binary residual feature fusion (BARF) and cross BARF. It extracts features from different deep learning methods and quantify the uncertainty of medical image classification results. Moloud Abdar et al.^52^ proposed a three-way decision-based Bayesian deep learning (TWDBDL) model to quantify the uncertainty in skin cancer image classification, avoids the loss of sensitive information. Mahesh Gour et al.^53^ proposed an uncertainty aware convolutional neural network model called UA ConvNet. The model applies MC dropout to forward propagation for obtain a posterior prediction distribution. By calculating mean and entropy, mean prediction and model uncertainty can be obtained. Alec Radford et al.^54^ considered transforming image classification tasks into image text matching tasks. They proposed the Comparative Text Image Training Model (CLIP), which predicts the correct matching relationship between images and text by jointly training image encoder and text encoder. Tan and Le^55^ proposed a convolutional neural network EfficientNet for image classification, which utilizes scaling of depth, width, and resolution to balance model complexity and accuracy. It adopts a Compound Scaling strategy to automatically adjust the size of the network, which can achieve better accuracy with relatively few computing resources.

### Discussion

CNNs have exhibited distinguished success on image classification tasks in recent years. Which essentially search for image template features with similar distributions in the image feature space to determine the category of input images. CNNs are crucial for extracting and representing iconic features of input images. However, existing distributed image representation learning methods and abstract high-level feature synthesis mechanisms still have limitations on highlighting the differences in local regional with global structural features between complex images^56^. For example, in medical images with amorphous structural boundaries, multiple organs and lesions may be intricately intertwined. This results in the ignorance of insufficient attention to local key features and potential saliency features, will reduce the generalization ability of classification models, along with the model robustness^57^. In addition, in the case of small sample set modeling, there still some difficulties such as lack of domain knowledge inspiration and constraint mechanisms, a large exploration space range, complex optimization and solution processes. These problems restrict the further improvement of the performance on complex images overall classification.

For medical image feature extraction, the recognition ability can effectively improve if the model can be guided to focus on local with global features unique to the image and embedded with prior knowledge of class features. Huang^58,59^ proposed a radial basis probabilistic neural network (RBPNN). It consists of an input layer, a radial basis probability neuron (RBPN) layer, a pattern aggregation layer and a classifier. Based on kernel function method, RBPNN fully considers the interleaving effect between multi class patterns in pattern recognition, which can form effective non-convex feature interfaces. It also contains fewer model parameters with low computational complexity. As the main information processing unit of RBPNN, RBPN adopts Gauss radial basis kernel function, whose adjustable variables are statistical parameters such as radial basis kernel center and variance. The radial basis kernel transform is a calculation of the distance between input data and kernel center, which is essentially a measure of the similarity of data features, providing an opportunity for the embedding of prior feature knowledge. If the action function of RBPN has probability significance, the probability output of image category attributes based on RBPN can be achieved.

## The methods and work proposed in this paper

In this paper, a ResNet-RBPNN model for whole image classification is proposed. It combines the feature extraction, representation mechanism of ResNet50 with the pattern class feature differentiation ability and class feature knowledge embedding mechanism from RBPNN. The first five layers of ResNet50 are used as visual modeling networks, utilizing feature pyramid convolution and pyramid pooling operations to enhance the scale invariance, in order to solve the problem of feature drift caused by changes in image perspective and target scale, achieve features extraction from targets with different sizes. Then, spatial and channel self-attention mechanisms are constructed to associate local and global image features. Feature fusion is achieved through skip-connections to extract unique features of the image, highlighting the significance of the global structural features of the image. In RBPNN-based classifiers, the RBPN layer is used to embed diversity class features. Based on the image features extracted by ResNet50, channel cosine similarity attention (CSA), which is insensitive to feature dimensions, is used to measure the similarity between input image features and radial basis kernel center image features. Dynamic C-means clustering algorithm is used to divide each image category into subcategories with more similar features, identifying typical samples of each subcategory with iconic class features, use these sample features as the kernel centers of each RBPN. An exponential sigmoid function with probabilistic membership properties is selected as kernel function to achieve the probability output of RBPN. In pattern aggregation layer, the output of the RBPN layer is selectively summed based on the class labeling of the kernel centers in each RBPN. Briefly, the integration involves consolidating the membership probabilities of input image features for subcategories into those for categories. This process merges the boundaries of each subcategory into non-convex boundaries of the category, thereby enhancing the discriminative power of image category features. The Softmax classifier utilizes the pattern layer’s output as input, implementing image classification based on the principle of maximum probability membership.

Medical images intuitively reflect the two-dimensional morphology and associated features of organs and tissues in specific areas within the human body. However, due to factors such as noise, bias deformation, grayscale distortion, and local positional effects, medical images often have complex content diversity and ambiguity. Meanwhile, since some diseases being uncommon in clinical practice, fewer samples were obtained. In addition, there are differences in anatomical structures of different individuals, which increase the difficulty of medical image features learning. Currently, deep learning has demonstrated significant advantages in image classification tasks, with numerous models proposed for image representation learning and classification. However, despite their impressive performance on natural images, these models typically have limitations on achieving comparable performance when applied to medical images. This is attributed to challenges such as indistinct morphological features. In this paper, ResNet-RBPNN is used to conduct classification experiments using common dataset of brain tumor MRI images and actual diagnostic dataset of cardiac system ultrasound images to verify the effectiveness of our method.

In the introduction and related work sections, we analyzed challenges in image classification tasks, as well as the current research status of deep learning for image classification and small sample set modeling. The idea and algorithm strategy based on prior feature knowledge embedding established in this paper are pointed out. In "ResNet-RBPNN image classification model" section, a deep learning model for image classification based on prior feature knowledge embedding was established, and its theoretical properties were analyzed. In "The experiment and result analysis" section, typical disease classification experiments and result analysis were conducted on two medical image datasets. Finally, the work of the paper is summarized, and the advantages and limitations of the proposed method are pointed out.

## ResNet-RBPNN image classification model

The ResNet-RBPNN proposed in this paper based on diversity prior category feature embedding mainly consists of two modules. The former is an image feature extraction and representation module based on the first 5 layers of the ResNet50. It employs feature pyramid convolution and pyramid pooling operations to extract various target features at multi-scales, and use channel-space mix attention to enhance the saliency of image-specific features. The latter is a radial basis probability neural network module to implement the embedding of prior category feature knowledge and image classification.

### The overall structure of ResNet-RBPNN

**Figure 1:**
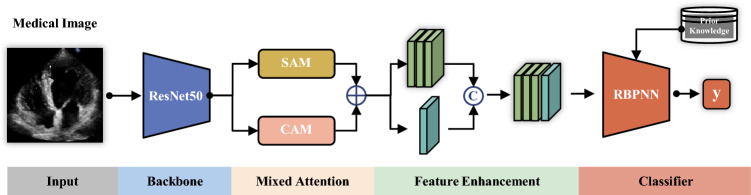
The ResNet-RBPNN image classification model.

The feature extraction and representation part of the ResNet-RBPNN includes image feature extraction based on ResNet50, information processing of spatial attention module (SAM) and channel attention module (CAM) mixed component, feature enhancement and representation units. RBPNN includes image category diversity prior feature embedding and image classification unit. The overall structure is shown in Fig. 1.

In Fig. 1, feature pyramid convolution (FPN) and pyramid pooling module (PPM) are used on first five convolutional layers of ResNet50 to obtain multi-scale feature maps of different receptive-fields, achieving the capture of feature information for various types of targets, especially small targets. On this basis, channel and spatial self-attention mechanisms are introduced. The spatial attention branch encodes broader spatial contextual information into local features to enhance their representation ability. Channel attention branching guides the network to focus on channels containing more feature information. In attention feature fusion and enhancement module, the aggregation mechanism of channel and spatial attention features is introduced to enhance the representation ability of the overall unique features of the image.

In the hidden layer of RBPN, prior feature of each class diversity is used as the kernel center of each RBPN to realize the embedding of prior feature knowledge in the model. In the pattern aggregation layer of RBPNN, the outputs of RBPN layer are selectively summed according to its kernel center category. That is, the category subcategory feature information is combined into category features to generate irregular class boundaries and improve the accuracy of image classification. Finally, the output of each node in the pattern aggregation layer is used as the input of the Softmax classifier to generate classification result.

### Image feature extraction based on multi-scale and attention mechanism

Feature pyramid convolution and pyramid pooling are used to build global scene prior information, extract and fuse multi-scale features with different receptive-fields. By constructing channel and spatial attention, as well as the fusion mechanism of channel and spatial features, the saliency of image global and local features is enhanced.

#### Multi-scale image feature extraction based on FPN and PPM

**Figure 2:**
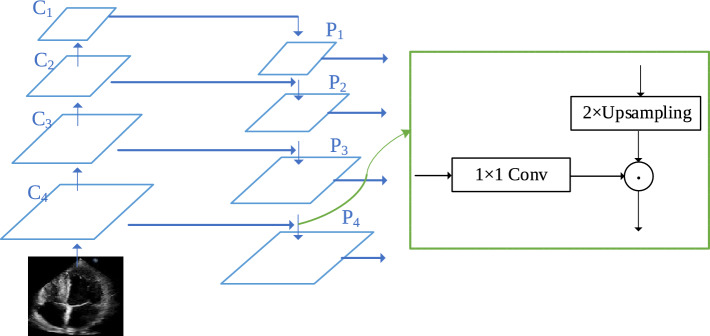
Feature pyramid network model.

FPN has a pyramid form of hierarchical feature representation, which can generate comprehensive features with strong semantic information at multi-scales. A top-down hierarchical structure and lateral connections are adopted to fuse shallow features with high-resolution and deep features with rich semantic information. The structure of FPN is shown in Fig. 2.

In Fig. 2, the input image is extracted based on Resnet50 to form a feature map of $C_4$ layer. Then, from bottom to top, through the $C_3$, $C_2$ and $C_1$ layers, convolution operations with a size of $3\times 3$ and a step size of 2 are performed in sequence, which is 1/2 downsampling to extract high-order semantic features of the image. Then, from top to bottom, bilinear interpolation upsampling is performed through the $P_1$, $P_2$, $P_3$ and $P_4$ layers to gradually restore the image size. Through lateral connection, shallow detail feature information and high-order semantic features are fused. The two layers of feature map with the same spatial size are connected horizontally, where $1\times 1$ conv is used to change the channel number of the feature map.

For the hierarchical extraction of image features, low-order features have rich detailed information, while high-order features contain more semantic information. FPN first upsamples the high-level feature map, then connects it laterally to previous layer based on same resolution to enhance the details in the high-level features.

**Figure 3:**
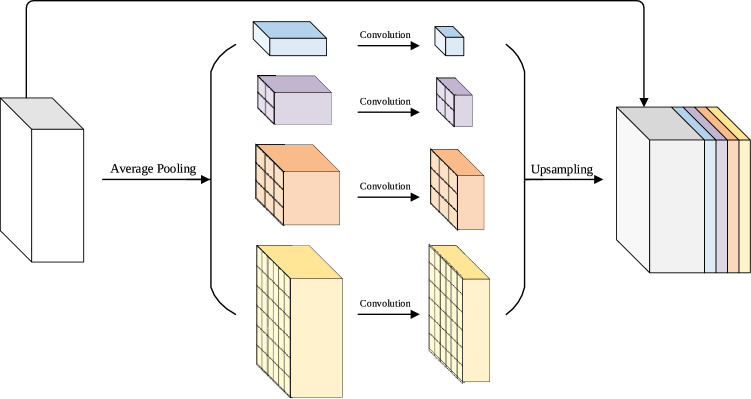
Pyramid Pooling Module(PPM) contains 4 different pyramid scales. The input feature maps are separately pooled to different target sizes, and the sizes of each layer are 1$\times$1,2$\times$2, 3$\times$3 and 6$\times$6 respectively.

PPM consists of a set of pooling blocks with different scales, which can better utilize global image prior knowledge to understand complex scenes, and extract features with global context information to improve image recognizability. It is a prior model that effectively associates global context, and its structure is shown in Fig. 3.

In Fig. 3, multi-scale pooling can preserve global context information at different scales. In order to maintain the weight of the global feature, a $1 \times 1$ convolution is performed on the pooled feature map, the number of channels is reduced to the original 1/*N* (In Fig. 3, N is 4). Then, low-dimensional feature maps are directly up-sampled by bilinear interpolation to obtain feature maps with the same size as the original feature maps. On this basis, original feature map and upsampled feature map are spliced according to the channel dimension, and the number of channels is doubled. Then use 1$\times$1 convolution to compress the number of channels to the original, finally obtain a feature map with the same size and number of channels as original feature map, which is used as output of the pyramid pooling module.

PPM is a hierarchical global prior structure, can build global scene prior information on the final layer feature map, reducing the loss of context information between different scales and sub-regions.

The first 5 convolutional blocks of ResNet50 used for visual modeling, $Conv_2$ to $Conv_5$ convolutional blocks use FPN, PPM for convolution and pooling operations. Thus, extract target image feature information with multi-scale.

#### Spatial and channel attention

For image classification tasks, because images have certain differences in scene perspective and appearance features, the model is required to have strong robustness and feature recognition capabilities. In practice, due to the limitation of the receptive field of the convolution operator, the model can only capture the information in the neighborhood, resulting in poor robustness when different images change in perspective and scale. The introduction of spatial attention can enable the network to learn how to leverage global context information to enhance the semantic features of the scene and each target. On the other hand, the richness of feature information contained in each channel of feature map is different. The introduction of channel attention can enhance or weaken the salience of each channel feature, guides the network to focus on channels containing more information.

**Figure 4:**
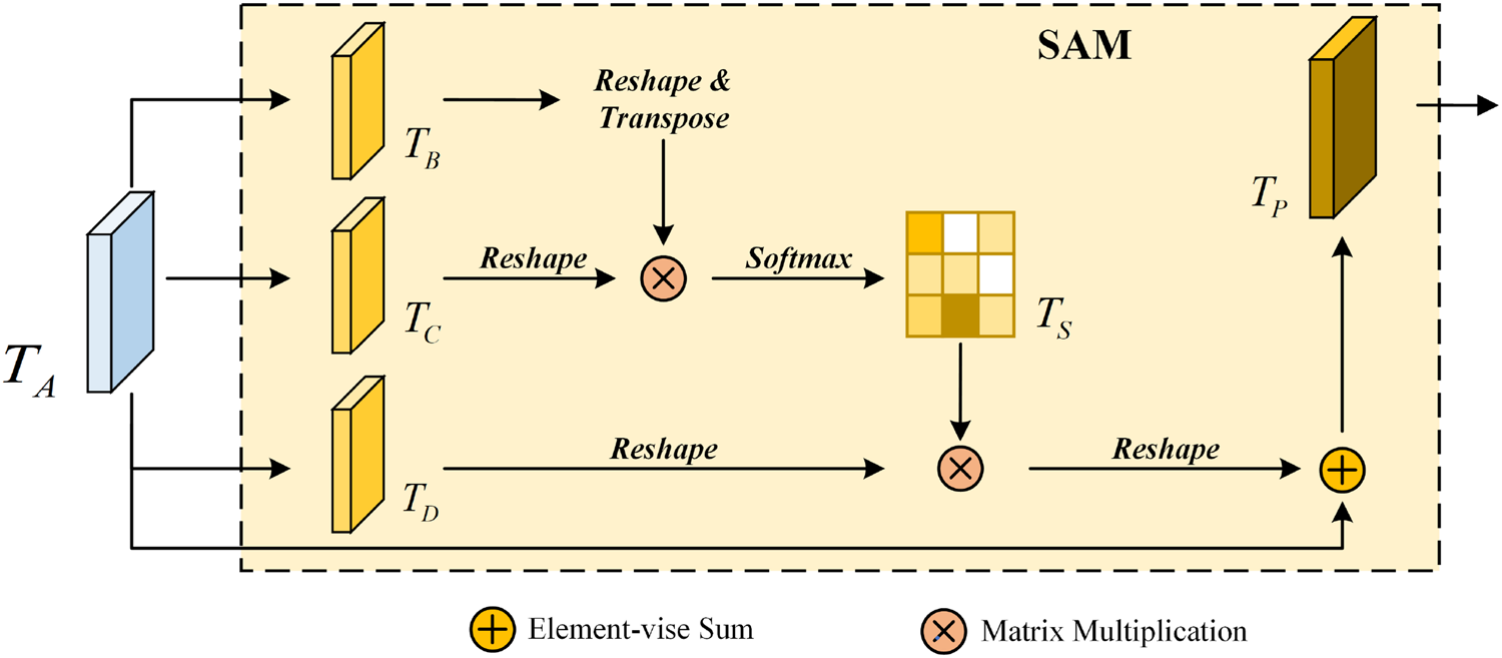
The structure of spatial self-attention module.

**Spatial attention mechanism.** The Spatial Attention Mechanism (SAM) employs attention to represent spatial information in order to encode broader contextual information into local features, thereby enhancing their representation capabilities. The structure of spatial attention module is shown in Fig. 4.

For a given input feature ${T_A} \in {{R}^{C \times H \times W}}$, 1x1 convolution is used for dimensionality reduction, and two new feature maps $T_B$ and $T_C$ are generated respectively, where $\left\{ {{T_B},{T_C}} \right\} \in {{R}^{C \times H \times W}}$. Then, reshape $T_B$ and $T_C$ into $R^{C\times \ HW}$. Reshape and transpose $T_B$ to obtain $T_B^T \in {{R}^{HW \times C}}$, then perform matrix multiplication with the reorganized $T_C$ to generate a spatial attention matrix, apply Softmax transformation to calculate the spatial attention map ${T_S} \in {\mathrm{{R}}^{HW \times HW}}$. This matrix models the spatial relationship between any two pixels of the feature map:

1 $$\begin{aligned} T_{S_{ji}}=\frac{exp(T_{B_i} T_{C_j})}{\sum _{i=1}^{N}{exp(T_{B_i} T_{C_j})}} \end{aligned}$$

where, $T_{S_{ji}}$ measures the impact of $i\text{- }th$ position feature on $j\text{- }th$. The more similar the feature representations of two locations, the stronger the correlation between them. $N = H \times W$, represents the number of pixels in the feature map.

In another pathway, feature $T_A$ is input to the convolutional layer to generate a new feature map ${T_D} \in {{R}^{C \times H \times W}}$ and reshaped into ${R ^ {C \times H \times W}}$. Then, a matrix multiplication is performed between $T_D$ and the spatial attention matrix $T_S$, the result is reshaped to obtain a tensor of dimension $C\times H \times W$. Finally, multiply the result matrix of the above multiplication by the scale parameter $\delta$, and perform element addition operation with the original feature $T_A$ to obtain the final output ${T_P} \in {R^{C \times H \times W}}$. The calculation process is as follows:

2 $$\begin{aligned} T_{P_j}=\delta \sum _{i=1}^{N}{\left( T_{S_{ji}}T_{D_i}\right) +T_{A_j}}. \end{aligned}$$

where, the scale parameter $\delta$ is initialized to 0 and gradually assigned larger weights through learning. $T_{P_j}$ represents each element in $T_P$.

From formula (2), $T_P$ is the weighted sum of the spatial attention feature map and the original features, based on supervised learning, the global context information of the target can be selectively aggregated.

**Figure 5:**
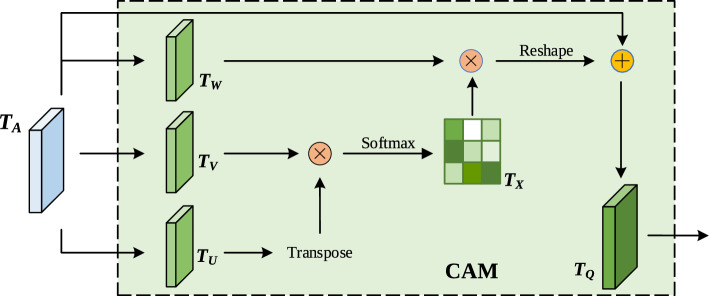
The channel attention module.

**The channel attention mechanism.** The channel attention mechanism (CAM) is used to model the interdependence between channels, and uses the interdependence between different channel feature maps to improve the feature representation of specific semantics, making the network focus on channels with larger weights. The structure of the channel attention module is shown in Fig. 5.

In Fig. 5, the given input feature ${T_A} \in {R^{C \times H \times W}}$ is first reshaped into $\left\{ {{T_U},\;{T_V}} \right\} \in {{R}^{C \times HW}}$, and then $T_U$ is transposed and matrix multiplied by $T_V$, and finally use the Softmax transformation to obtain the channel attention map ${T_X} \in {{R}^{C \times C}}$:

3 $$\begin{aligned} T_{X_{ji}}=\frac{exp(T_{U_i} T_{V_j})}{\sum _{i=1}^{C}{exp(T_{U_i} T_{V_j})}}. \end{aligned}$$

$T_{X_{ij}}$ measures the influence of the $i\text{- }th$ channel on $j\text{- }th$.

At the same time, reshape $T_A$ again to get ${T_W} \in {{R}^{C \times HW}}$, perform matrix multiplication between $T_X$ and $T_W$, and reshape the result ${R^{C \times HW}}$ into ${R^{C \times H \times W}}$. Then, the result is multiplied by the scale parameter $\in$, and an element-wise sum operation is performed with $T_A$ to obtain the final output ${T_Q} \in {{R}^{C \times H \times W}}$.

The specific calculation is as follows:

4 $$\begin{aligned} T_{Q_j}=\epsilon \sum _{i=1}^{C}{\left( T_{X_{ji}}T_{W_i}\right) +T_{A_j}}. \end{aligned}$$

where, the scale parameter $\epsilon$ is initialized to 0 and gradually assigned larger weights through learning. $T_{Q_{j}}$ represents each element in $T_Q$, *C* represents the number of channels of the input feature.

From Eq. (4), the final feature of each channel is the weighted sum of features from all channels and the original features, thus establishing a global semantic dependency between feature maps.

**Figure 6:**
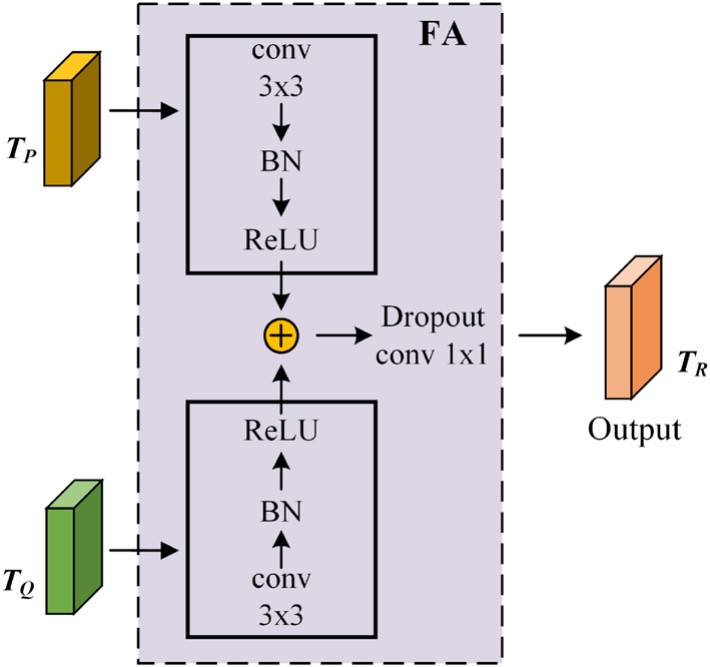
The SAM and CAM aggregation module.

**Channel and spatial attention feature aggregation.** To fully utilize the information in spatial and channel dimensions and enable the model automatically focus on important spatial regions, we parallel-process the output features of the $Block_3$ layer in ResNet50 with SAM and CAM. For the output $T_P$ and $T_Q$ of the two modules, 3$\times$3 convolution, batch normalization (BN) and activation function ReLU are used respectively to improve the nonlinear expression ability of the network. Then, use the element addition operation to aggregate the processed features, perform Dropout and 1$\times$1 convolution on the aggregated features to prevent the network from over-fitting during training stage. The whole hybrid attention module finally outputs aggregated features $T_R$. Its implementation process is shown in Fig. 6.

The calculation process is as follows:

5 $$\begin{aligned} T_R=\eta \left( \mu \left( SAM\left( T_A\right) \right) +v\left( CAM(T_A)\right) \right) . \end{aligned}$$

where, both $\mu$ and $\nu$ represent 3$\times$3 convolution, BN and ReLU processing, and $\eta$ represents Dropout and 1$\times$1 convolution. $SAM\left( T_A\right)$ represents spatial attention calculation, $CAM\left( T_A\right)$ represents channel attention calculation.

The aggregated feature $T_R$, that is, the final output in Fig. 6, is applied to subsequent operations, therefore network can adaptively focus on important areas and channels. Finally, the image features aggregated by channel and spatial features will be integrated to generate a comprehensive feature matrix *F* with multi-scale and feature enhancement.

6 $$\begin{aligned} F = \left[ \begin{array}{l} {h_{11}}\;\;{h_{12}}\;...\;\;{h_{1L}}\\ {h_{21}}\;\;{h_{22}}\;...\;\;{h_{2L}}\\ ...\;\;...\;...\;...\;\;\\ {h_{n1}}\;\;{h_{n2}}\;...\;\;{h_{nL}}. \end{array} \right] \end{aligned}$$

In subsequent processing, the matrix shown in formula (6) is used to represent the comprehensive features of each image in sample set.

### Category prior feature embedding based on RBPNN

**Figure 7:**
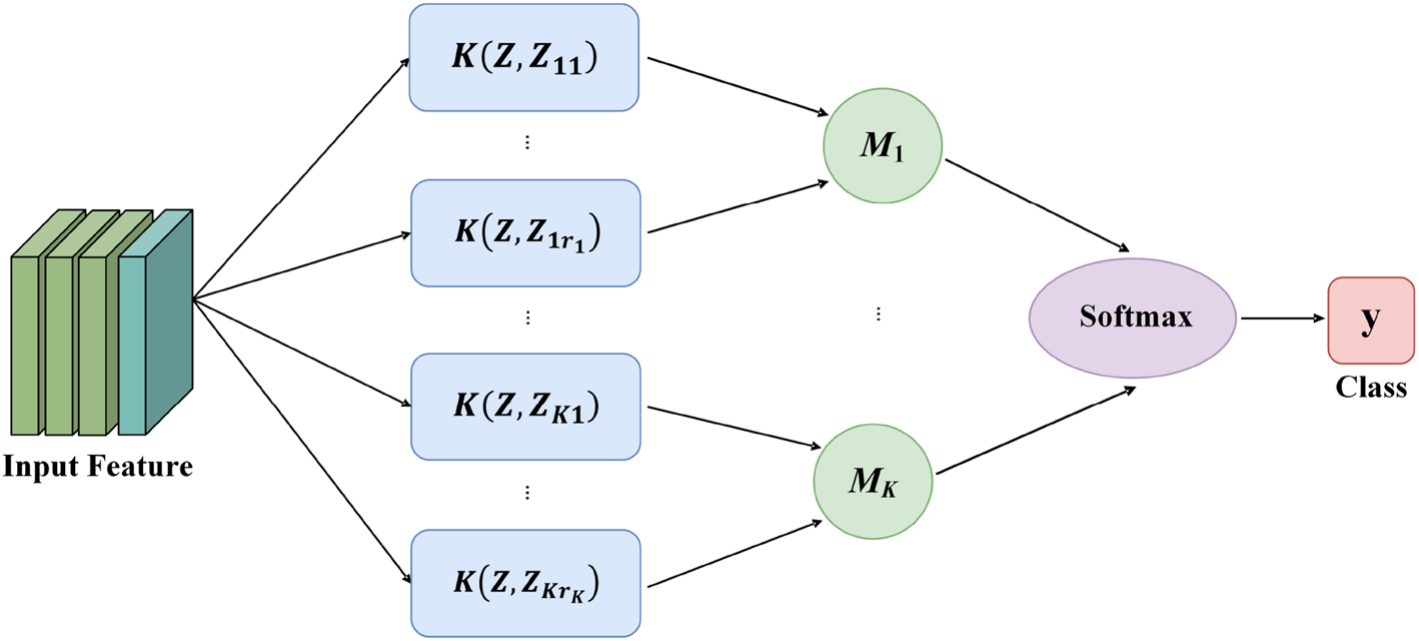
The structure of RBPNN.

Based on the comprehensive feature extraction and representation of the whole image, a radial basis probabilistic neural network (RBPNN) for prior image feature knowledge embedding is established. It consists of an image synthesis feature input layer, a Radial Basis Probabilistic Neuron (RBPN) layer, a pattern aggregation layer, and a Softmax classifier. Its structure is shown in Fig. 7.

In Fig. 7, $K(\cdot ,\cdot )$ is the radial basis kernel function, and *Z* is the input image feature. $Z_{ki}(k=1,2,\cdots ,K;i=1.2.\cdots ,C_k)$ represents the radial basis kernel center, where *K* represents the number of pattern categories, and $C_k$ represents the number of typical image features selected in the $k\text{- }th$ category. $M_1,M_2,\cdots ,M_K$ represent the pattern aggregation operators.

Figure 7 shows the information processing process of RBPNN and the corresponding relationships between input and output of each layer are established. In the input unit of the model, the image feature matrix extracted by ResNet is input. A one-dimensional feature vector is generated through feature reorganization and passed to the RBPN layer. In RBPN layer, the diversity typical features selected by the DCM algorithm for each category are used as the kernel center of each RBPN to achieve the embedding of feature knowledge. The channel cosine similarity attention (CSA) algorithm is used to measure the similarity between the input image features and the RBPN kernel center features, and the radial basis kernel function is used to stimulate the output. In RBPNN pattern aggregation layer, the outputs of the RBPN layer are selectively summed according to the category of kernel center, which combines the feature information of class subclasses into class features to generate irregular class boundaries. Finally, the output of each node in the pattern aggregation layer is used as input of the Softmax classifier to achieve image classification.

#### The selection of typical features for image categories

In image sample set, due to differences in scene perspectives, target appearance features, and spatial relationships among different images, samples in the same image category often exhibit diverse local and global features. In this paper, a CSA based image similarity measurement mechanism and DCM algorithm are used to divide each image category into several subclasses with more similar global and local features. The clustering centers of each subcategory are selected as representative features, implicitly representing the diversity of image category features.

**Image channels cosine similarity measurement.** The image samples features are extracted through ResNet to obtain multi-channel feature information. The feature maps of different channels are reorganized into feature vectors, and multi-channel feature maps are used to form a feature matrix. Calculate the cosine distance between the row vectors of each feature matrix to define the similarity between the two feature matrices. The calculation formula is as follows^60^:

7 $$\begin{aligned} \cos \left( {A,B} \right) = \frac{{A \cdot B}}{{{{\left\| A \right\| }_2}{{\left\| B \right\| }_2}}} = \frac{{\sum \limits _{i = 1}^n {\left( {{x_i} \times {y_i}} \right) } }}{{\sqrt{\sum \limits _{i = 1}^n {{{\left( {{x_i}} \right) }^2}} } \times \sqrt{\sum \limits _{i = 1}^n {{{\left( {{y_i}} \right) }^2}} } }}. \end{aligned}$$

where, the smaller the cosine distance between two vectors A and B, the higher similarity between vectors A and B. Conversely, the larger the cosine distance, the lower similarity between A and B.

Cosine similarity is used to describe the similarity of different channel feature of the feature map X, and a similarity matrix M with a dimension of $C \times C$ is obtained^61^. The calculation formula is as follows:

8 $$\begin{aligned} {M_{ij}} = \cos \left( {{F_i},{F_j}} \right) . \end{aligned}$$

where, $F_i$ and $F_j$ represent the feature vectors of different channels in the feature map. Normalize the elements of each row of vectors in M, then calculate the average value to represent the overall similarity between this channel and other channel features, denoted as $M_C$.

**Channel cosine similarity attention.** CSA not only considers the similarity between different channels in the feature map, but also introduces an attention mechanism to the channel. It represents the importance of the channel feature in feature map by defining a weight^62^. The features attention coefficient $A_c$ of channel *c* is calculated based on average pooling, and a channel weight vector $A_C$ with a dimension of $1 \times 1 \times C$ is obtained. The calculation formula is as follows:

9 $$\begin{aligned} {A_c} = \frac{1}{{H \times W}}\sum \limits _{i = 1}^H {\sum \limits _{j = 1}^W {{x_{ij}}} }. \end{aligned}$$

where, $x_{ij}$ is the eigenvalue of row *i* and column *j* in feature map X.

The feature information is fused by the attention coefficient and feature similarity matrix, the c-channel of the feature map is multiplied to obtain the fused features. The calculation formula is:

10 $$\begin{aligned} {F_w}\left( {{X_c},{M_c},{A_c}} \right) = {X_c} \cdot {M_c} \cdot {A_c}. \end{aligned}$$

where, $X_c$ is the feature vector of the $c\text{- }th$ channel in the feature map *X*, and $F_w$ represents the fusion operation.

Image features are processed using CSA. The similarity of salient features between images can be calculated more accurately based on the weights of channel features.

#### Selection of pattern classes typical feature samples

For the embedding of prior category feature knowledge, the selection of diversity typical image feature templates of each category will directly affect the classification results. In this section, based on the image sample set and the CSA image feature similarity measure, the dynamic C-means clustering algorithm (DCM) is used to select typical features and form a feature template set.

The DCM algorithm is a dataset partitioning method that obtains the feature similarity of each sample point to all cluster centers through objective function optimization, and then determines the class membership of the sample points. In this section, a dynamic C-means clustering algorithm^63^ is introduced to address issues such as difficulty in determining the number of clusters in advance and sensitivity to initial values in the C-means clustering algorithm. Firstly, set different clustering numbers and calculate the coupling degree and separation degree between image sample features based on CSA similarity. Evaluate the clustering results corresponding to different number of clusters, select the optimal clustering result, and determine the optimal number of clusters.

Assuming the dataset contains N image samples, and the density at the sample point feature $X_i$ is defined as^64^:

11 $$\begin{aligned} {D_i} = \sum \limits _{k = 1}^n {\frac{1}{{1 + {f_d}||{X_i} - {X_k}||_F^2}}} \mathrm{{ }}, i = 1,2, \cdots ,N \end{aligned}$$

where, $|| \cdot ||_F^2$ is the distance norm induced by the similarity measurement algorithm, and ${f_d} = 4/{r_d}^2$, ${r_d} = \frac{1}{2}\sqrt{\frac{1}{{N(N - 1)}}\sum \limits _{k = 1}^N {\sum \limits _{i = 1}^N {||{X_i} - {X_k}||_F^2} } }$ is the effective radius of the neighborhood density. The larger the ${D_i}$ , the denser the sample points around ${X_i}$ , and the greater the density at ${X_i}$.

We calculate the density of each sample feature point in sample set, delete the points in low-density area, and obtain the sample feature set *D* in the high-density area. Select the sample feature point with the highest density as the first cluster center $Z_1$ , then select the sample feature point farthest from $Z_1$ as the second cluster center $Z_2$. Then, calculate the distances between remaining sample feature points and $Z_1$, also $Z_2$. Chose the sample feature point with the maximum value as the third cluster center $Z_3$. Repeat like this to find *C* initial cluster centers.

We pre-set different clustering numbers for clustering, the clustering results correspond to different divisions of sample feature sets. The segmentation results are evaluated by the coupling degree and separation degree between sample features^65^. Here, the coupling degree is defined as:

12 $$\begin{aligned} {C_d}(c) = \frac{1}{n}\sum \limits _{k = 1}^c {\sum \limits _{i = 1}^N {\mu _{ik}^md_{ik}^2} \mathrm{{ }}};k = 1,2, \cdots ,c,i = 1,2, \cdots ,N\mathrm{{ }} \end{aligned}$$

The separation degree is defined as:

13 $$\begin{aligned} {S_d}(c) = \frac{{\sum \limits _{i,k = 1;i \ne k}^c {d_{ik}^2} }}{{[c(c - 1)]/2}}\mathrm{{ }};i,k = 1,2, \cdots ,c\mathrm{{ }} \end{aligned}$$

The coupling degree represents the compactness within the class, and the smaller it is, the better the compactness. The separation degree represents the separation between classes, and the larger it is, the higher the separation.

The index *GD*(*c*) is used to evaluate the clustering results:

14 $$\begin{aligned} GD(c) = \alpha {C_d}(c) + (1 - \alpha )\frac{1}{{{S_d}(c)}} \end{aligned}$$

where, $\alpha$ is the coupling weight factor.

For different clustering numbers, a smaller *GD*(*c*) represents a better clustering result. The *C* value corresponding to the minimum value of *GD*(*c*) is the optimal clustering number, and the division of sample feature set corresponds to the optimal clustering. The center of each cluster is chose as the typical image feature of the sample set.

### RBPNN classification based on image features

Using image synthesis features as the input of RBPN and exponential sigmoid as the kernel function of RBPN, the similarity between input image features and the RBPN kernel center is calculated based on CSA. By selectively aggregating the membership probabilities of each subcategory through the pattern aggregation layer, the input image is classified based on Softmax.

**The division of pattern subclasses and the determination of RBPN kernel centers.** There are *K* categories ${C_k}(k = 1,2, \cdots ,K)$ in training set. $C_k$ contains $m_k$ typical sample category features, which are respectively recorded as ${Z_{kl}}\;(l = 1,2, \cdots ,{m_k})$. The number of nodes in the RBPN layer is $m = {m_1} + {m_2} + \cdots + {m_K}$.

In various subsets, CSA is used to measure the similarity between sample features, DCM algorithm is used to partition clustering subsets, and each clustering center is selected as a typical image feature for that category. A total of *m* typical sample features is selected in the sample set, arranged in order as ${z_{11}}, \cdots ,{z_{1{m_1}}}, \cdots ,{z_{21}}, \cdots ,{z_{2{m_2}}}, \cdots ,\;{z_{K1}}, \cdots ,{z_{K{m_K}}}$. The first subscript of $z_{kl}$ represents category *k*, and the second subscript represents the ordinal number of the $l\text{- }th$ typical sample feature in $k\text{- }th$ classification. $z_{kl}$ is sequentially used as the kernel center of each node in the RBPN layer. According to class identifier *k* of $z_{kl}$, the outputs of RBPN are selectively summed towards the pattern aggregation layer node.

Assuming that the comprehensive feature of the input image *X* is $F_X$, and RBPN kernel function is an exponential sigmoid function, the calculation formula for the output of RBPN layer nodes is:

15 $$\begin{aligned} h_{kl}=\frac{1}{1+\exp {\left( -a\left( \frac{-CAS\left( F_X-z_{kl}\right) }{\sigma _k^2}\right) -c\right) }} \end{aligned}$$

where, $\sigma _k$ is the sample feature variance for the $k\text{- }th$ category, *a* and *c* are smoothing parameters.

The output of the pattern aggregation layer is:

16 $$\begin{aligned} q_k=\sum _{j\in \Omega _k} h_{kl}\;\;\;(k = 1,\;2,\; \cdots ,\;K) \end{aligned}$$

where, $\Omega _k$ is the set of RBPN layer node numbers whose kernel center belongs to class *k*. The probability of Softmax classifier categorizing input image *X* into $k\text{- }th$ class is:

17 $$\begin{aligned} p=\left( y=k| X;\theta \right) =\frac{e^{\theta _k^T\cdot q}}{\sum _{k=1}^{K}e^{\theta _k^T\cdot q}} \end{aligned}$$

$q = (q_1, q_2, \cdots ,q_K)$ is the output of the pattern aggregation layer, $\theta = ({\theta _1},{\theta _2}, \cdots ,{\theta _K}),{\theta _l} = ({v_{1l}},{v_{2l}}, \cdots ,{v_{Kl}}),l = 1,2 \cdots ,K$. ${v_{kl}}$ is the connection weight from $k\text{- }th$ node of the pattern layer to $l\text{- }th$ input node of softmax.

According to the principle of maximum probability output, the classification result of the input image is:

18 $$\begin{aligned} y=argmax\left( y=k| X;\theta \right) \end{aligned}$$

## The experiment and result analysis

### The classification experiment based on the common dataset of brain tumor MRI images

Brain tumor is an invasive disease, with the help of magnetic resonance imaging (MRI), doctors can obtain information about the location, size, and type of tumors for diagnosis and subsequent treatment plans. The Brain Tumor Classification dataset image samples are generated based on MRI scans and were established by Bhuvaj team in 2020^66^. The dataset includes a total of 3264 images, including four labels: glioma, meningioma, pituitary adenoma and no tumor. Among them, 2870 images are in the training set and 394 images are selected as test set. The sample distribution of the dataset is shown in Table 1.

**Table 1:** Distribution of experimental sample data.

Type		Training set	Test set	Total	Label	
Glioma tumor		826	100	926	0	
Meningioma tumor		822	115	937	1	
Pituitary tumor		827	74	901	2	
No tumor		395	105	500	3	
Total		2870	394	3264		

Three mainstream deep learning image classification models that can directly perform image classification were selected: MOCOv2^37^, DINOv2^38^, EfficientNet-v2^55^ for comparative experiments. The training and testing sets shown in Table 1 are used for disease classification diagnosis.

In the experiment, the hardware environment built is GPU: RTX3070, with video memory GDDR6, and video memory size is 10GB; CPU: Intel Core i9-12900K, with 16 cores and 24 threads, base frequency 3.2 GHz, and memory size is 32GB.

Comparing model architectures. DINO v2 uses the Vision Transformer (ViT-S/14) as backbone, stacking five Transformer encoder layers, each containing self-attention and feedforward networks, with Layer-Norm used for normalization between layers. The model has a learnable parameter count of 67*M*. MoCo v2 consists of two input channels, with the first channel consisting of a query encoder and a two-layer MLP projection head, using ResNet 50 as the encoding network. The second channel consists of a momentum encoder and a 2-layer MLP projection head shared with the parameters of the first channel. The momentum encoder is obtained by exponentially moving the query encoder. This model is pretrained through contrastive learning based on the output results of two channels, then fix the encoder parameters and use Softmax for classification. The model has a learnable parameter count of 25*M*. The EfficientNet v2 model consists of 42 stacked convolutional layers, including one $3\times 3$ convolutional layer, 10 depthwise separable convolution and pointwise convolution fusion layers (Fused MBConv), 30 MBConv layers, and 1 Conv$1\times 1$ &Pooling &FC module. Each Fused MBConv layer introduces SENet with a scaling factor of 0.25. The model has a learnable parameter count of 22*M*. The proposed model in this paper mainly consists of a feature extraction module based on ResNet 50, a spatial and channel mixed attention module, a feature enhancement module, and a diversity feature knowledge embedding and classification module based on RBPNN. The number of learnable parameters is 14*M*.

Image preprocessing. (1) Resize: Use transforms.Resize(224, 224) to resize all images to $224\times 224$; use transforms.ToTensor() to convert the images to tensors. (2) Normalize: Use transforms. Normalize(mean=[0.485, 0.456, 0.406], std=[0.229, 0.224, 0.225]) to normalize the image values to between 0 and 1.

Experimental hyperparameter settings. All three comparison models use the Adam optimizer, with hyperparameters set to: epochs=20, learning rate=0.0001, decay coefficient=0.1, and loss function is set to CrossEntropyLoss.

For each type of brain tumor, the DCM algorithm is used to divide the category into several image subcategories with more similar features. Specifically, 55 subcategories of gliomas, 40 subcategories of meningiomas, 30 subcategories of pituitary tumors, and 20 subcategories without tumors were identified. Using the clustering centers of each subclass as typical sample features, form a set of diversity prior feature templates. The model structure parameter setting and processing flow of this paper are as follows: one node in the input layer, corresponding to the input of image samples. After processing by the image feature extraction module, mixed attention and feature enhancement module, and feature aggregation module, the significant features of image samples are generated. The RBPN layer is divided into four groups, with the kernel centers corresponding to 55, 40, 30, and 20 typical sample features in four categories; the pattern aggregation layer has four nodes, corresponding to four types of diseases. The Softmax classifier takes the output of the pattern layer as input to achieve classification discrimination.

The performance evaluation indicators of the classification model are accuracy, recall, precision and F1-Score. The experimental results and training time are shown in Table 2.

**Table 2:** The classification results and evaluation indicators of proposed method.

Model	Accuracy (%)	Recall (%)	Precision (%)	F1-Score (%)	Training time(s)
DINOv2	80.8	85.09	79.83	82.38	6684.548
MOCOv2	75.38	80.21	73.77	76.85	5414.484
EfficientNet-v2	78.43	85.14	76.8	80.75	2807.350
The proposed	85.28	88.12	84.03	86.02	823.014

According to Table 2, the proposed method has achieved best results in all evaluation indicators. The main reason is that the morphological characteristics of brain tumors are not significant, the lesion target is small and strongly correlated with other organs, the signal-to-noise ratio is low, and there are situations of “One disease with multiple symptoms, and Multiple diseases with the same symptom” in medical. The proposed method can embed and memorize prior class features of different disease diversity, as well as achieve classification based on non-convex class boundaries, improving robustness and generalization ability. In terms of training time, EfficientNet v2 has the highest computational efficiency among the three compared models. However, due to its simpler structure, the training time of the model in this paper is 823.014s. However, the preprocessing time for selecting diverse representative sample features for each category is relatively long, and the DCM algorithm runs for 1532.421s. Therefore, the total training time for this algorithm is 2355.435s.

**Figure 8:**
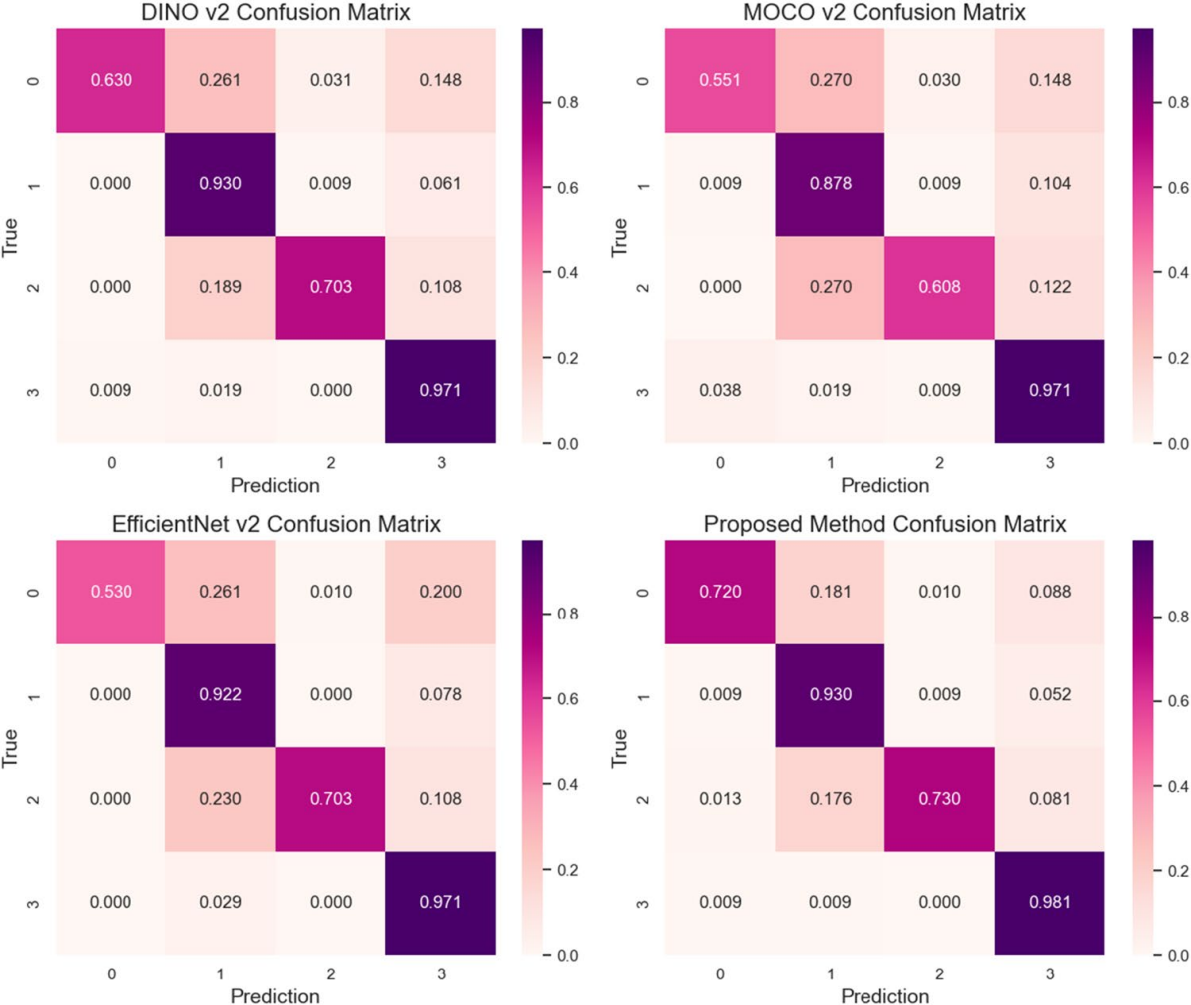
Four methods confuse matrices.

Calculate the confusion matrix based on the classification results on the test set, and compare the discriminative ability of four methods for samples with similar features in different categories. The results are shown in Fig. 8.

From the confusion matrices in Fig. 8, it shows all methods have lower recognition rates for gliomas compared to other diseases, as well as poorer robustness and generalization ability. However, compared to the other three models, the performance evaluation indicators of the proposed method have significantly improved, indicating that the proposed method can effectively enhances the saliency of local features in images and improve the discrimination ability for category feature differences.

### The classification experiment based on small sample dataset of cardiac ultrasound images

#### Dataset

This experimental dataset includes 2D echocardiography images in the apical four chamber view and diagnostic results reports from 928 hospitalized patients and physical examination personnel in a certain hospital. The resolution of ultrasound images is 224 $\times$ 224. It includes four types: type II diabetes mellitus with coronary heart disease (T2DM with CHD), pure CHD, non-CHD heart disease, and normal. Different types have different pathological characteristics. We select ultrasound images of the late diastolic and late systolic stages of the heart to form a single sample, in order to implicitly compare the changes in image features at these two moments and increase the feature information for disease diagnosis. Due to the relatively fixed position and spatial structure of various organ tissues, the two images were concatenated and synthesized into one image. During the treatment process, the focus is on the morphology and changes of target structures. These structures include the left ventricle, mitral valve, and ascending aorta. The goal is to analyze the characteristics of myocardial function, blood supply, and ejection function, as well as structural changes. Additionally, disease classification diagnosis is performed. The distribution of experimental sample data is shown in Table 3.

**Table 3:** Distribution of experimental sample data.

Model		Quantity	Training set	Test set	
T2DM with CHD		221	154	67	
Pure CHD		250	175	75	
Non-CHD		233	163	70	
Normal		224	168	56	
Total		928	660	268	

**Figure 9:**
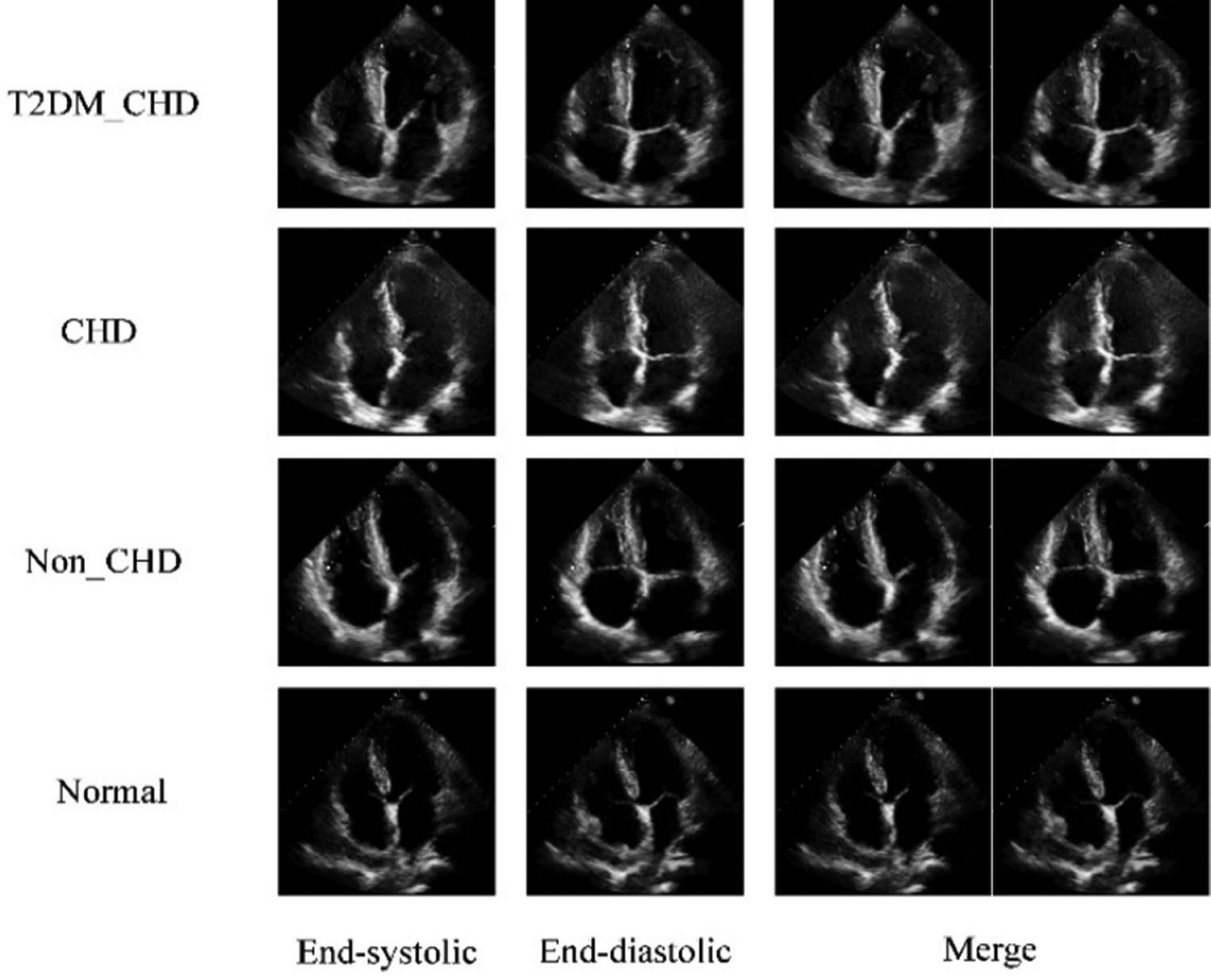
Typical ultrasound images of 4 types of diseases.

The ultrasound images of four types of diseases in late systolic, late diastolic, and concatenated images are shown in Fig. 9.

#### The construction of a typical image feature template set

For each category, considering the differences between sample individuals and the diversity of disease features within the same category, DCM algorithm is applied to finely divide the class sample subset into several pattern subclasses with more similar features. In the experiment, 37 subcategories in T2DM with CHD, 30 subcategories in pure CHD, 30 subcategories in non-CHD, and 20 subcategories in normal were identified. The clustering centers of each pattern subclass are used as typical sample features to form a diversity prior feature template set.

#### Experiment and result analysis

The ResNet-RBPNN model used for coronary heart disease classification is shown in Fig. 1, and the structural parameter settings and processing process are as follows. One node in the input layer corresponds to one image sample input. After processing with the image feature extraction module, mixed-attention feature enhancement module and feature aggregation module, local and global unique saliency features of the image samples are generated. The RBPN layer is divided into four groups, with kernel centers corresponding to 37, 30, 30, and 20 typical sample features of the four types of patterns. The pattern aggregation layer has 4 nodes corresponding to four types of coronary heart disease. The Softmax classifier uses the output of the pattern layer as input to achieve classification.

The sample set was randomly divided into two groups according to pathological proportions, with one group consisting of 660 samples forming the training set and the other group consisting of 268 samples forming the test set. The Mini Batch Gradient Descent (MBGD) algorithm is used for model parameter training. The training error accuracy is set to 0.05, the maximum number of training epochs is 10000, the learning rate is set to 0.01, batch$\_$size = 10, epoch = 20. Accuracy, recall, precision, and F1-score are used as performance evaluation indicators for classification models. Perform classification on the test set, and the classification results and various evaluation indicators are shown in Table 4.

**Table 4:** Experimental results of proposed method.

Model		Accuracy(%)	Recall (%)	Precision (%)	F1-Score (%)	
T2DM with CHD		75.63	72.86	76.12	74.45	
Pure CHD		76.84	73.42	77.33	75.32	
Non-CHD		76.65	84.38	77.14	80.6	
Normal		88.8	90.91	89.29	90.09	

According to Table 4, the proposed method achieved better results. This is because for medical image-based disease diagnosis, the proposed method embeds diverse typical category feature knowledge of four diseases into the model, effectively implementing model and data constraints, strengthening and maintaining the diversity of pattern features, and reducing the requirement for sample set completeness. In the case of small-scale sample sets, this ensures the robustness and generalization ability of the model.

Ablation experiments. To verify the effect of the feature pyramid convolutional pyramid, pyramid pooling module, SAM and CAM hybrid attention module, feature enhancement module, and prior feature knowledge embedding module on the classification performance of the model, comparative experiments were designed in which each module was ablated separately. Each experiment removed one module to evaluate the role of different components. The experimental results are shown in Table 5.

**Table 5:** The result of ablation experiments.

FPN& PPM	SAM& CAM	Feature enhancement	Knowledge embedding	Accuracy (%)	Recall (%)	Precision (%)	F1-Score
✕	$\checkmark$	$\checkmark$	$\checkmark$	73.33	74.06	73.95	74.01
$\checkmark$	✕	$\checkmark$	$\checkmark$	76.49	77.08	77.10	77.09
$\checkmark$	$\checkmark$	✕	$\checkmark$	77.61	78.32	78.16	78.24
$\checkmark$	$\checkmark$	$\checkmark$	✕	69.03	69.50	69.68	69.59
$\checkmark$	$\checkmark$	$\checkmark$	$\checkmark$	83.92	84.54	84.33	84.44

From Table 5, it can be seen that the FPN &PPM module has a significant impact on the classification results. Removing is significantly reduces the performance evaluation indicators of the model, reducing the model’s ability to capture various objects with large differences in shape and size in the image. If the SAM and CAM hybrid attention module is removed, it weakens the model’s ability to enhance the contrast and boundary features between objects with different shapes and sizes in medical images, resulting in a decrease in model performance indicators. If the feature enhancement module is removed, it weakens the model’s ability to fuse low-level features with high-order semantic features, as well as its ability to represent subtle morphological and structural features of various objects in the image. The feature knowledge embedding module has the greatest impact on classification results. Removing it significantly reduces the model’s classification ability, generalization ability, robustness, and interpretability.

Comparative experiment. In the comparative experiment, DINOv2, MOCOv2 and EfficientNet-v2 were selected for comparison. Four methods are used for disease classification based on the same training and testing sets.

In the experiment, a 4-fold crossover scheme was applied. The sample set was randomly divided into four equal groups according to the proportion of disease types. Three of them were combined to form a training set, and one group was used as a test set for four experiments. The average value of various evaluation indicators based on the results of four experiments is used as a comparative indicator.

**Table 6:** The results of comparison methods classification.

Model		Accuracy(%)	Recall (%)	Precision (%)	F1-Score (%)	
DINOv2		75.06	75.34	75.94	75.64	
MOCOv2		72.1	73.14	72.74	72.94	
EfficientNet-v2		76.53	77.66	76.92	77.29	
The proposed		83.92	84.54	84.33	84.44	

According to Table 6, the proposed method achieved the best results in all evaluation indicators. This is due to the embedding of prior knowledge of diversity class features and the classification decision of non-convex class boundaries, which improves the model’s ability to distinguish class feature differences and generalization. As three “end-to-end” deep models, MOCOv2, DINOv2, and EfficientNet-v2 have numerous parameters and require large-scale and complete sample sets to support the model. In the case of incomplete and small-scale imbalanced datasets, excessive freedom in structure and parameter selection reduces the robustness and generalization ability of the model. Meanwhile, the samples in the dataset come from real cases in hospitals, and the data quality and annotation accuracy are not as good as standard datasets and public datasets, which also leads to poor generalization ability of three comparison models. The proposed method has better robustness and generalization ability due to its targeted functional module design targeting image characteristics.

## Conclusion

In this paper, a deep learning model based on embedding prior class feature knowledge is established for complex image classification with insignificant morphological structures and low signal-to-noise ratio and small sample set modeling. It has designed targeted functional modules based on image characteristics, which can effectively improve the extraction performance of unique features for medical images. At the same time, based on RBPNN, the embedding of diverse prior feature knowledge is achieved, which can form complex classification boundaries and improve the recognition ability and robustness of the classification model. For the proposed method, the completeness of the image category feature template set has a significant impact on the classification results. However, for large-scale sample sets, with high construction workload of template sets and significant computational complexity, in some cases, manual participation is required. In this comparative experiment, MoCo and DINO are unsupervised image classification models. Further research is needed to compare the differences and commonalities between supervised and unsupervised learning mechanisms in application, as well as their applicable conditions. Meanwhile, method validation should be considered for long tailed distribution datasets to better reflect real-world scenarios. These issues will be the work that we need to solve in next stage. Applying ResNet-RBPNN to disease classification in the common dataset of brain tumor MRI images, with significant improvements in various performance evaluation indicators. For the classification of small sample sets of coronary heart disease, compared with the current three mainstream models, it has improved the accuracy by 8.84%, recall rate by 6.88%, accuracy by 7.41% and F1 Score by 7.15%. These indicate that the proposed method has strong ability to extract and identify local and global features in medical images, and has a good application potential.

## Data Availability

Our paper incorporates experiments using both public and non-public datasets. The non-public dataset, comprising retrospective clinical data, is sourced from the Affiliated Hospital of Qingdao University. This dataset has undergone thorough scrutiny by the Medical Ethics Committee, emphasizing our commitment to the ethical use of medical data. We don’t have permission to publish the dataset. Any questions, please contact the author Chen Xu by c20_xu@fudan.edu.cn. For public datasets, you can access the resource by https://www.kaggle.com/datasets/sartajbhuvaji/brain-tumor-classification-mri, the dataset author is Sartaj Bhuvaji and his team.
